# Advances in the diagnosis, immunopathogenesis and therapies of IgM-anti-MAG antibody-mediated neuropathies

**DOI:** 10.1177/1756285617746640

**Published:** 2018-01-15

**Authors:** Marinos C. Dalakas

**Affiliations:** Neuroimmunology Unit, Department of Pathophysiology, National and Kapodistrian University of Athens Medical School, Athens, Greece

**Keywords:** polyneuropathy, anti-MAG antibodies, autoimmune neuropathies, neuropathy with monoclonal gammopathy, rituximab, anti-B cell therapies

## Abstract

Polyneuropathy with immunoglobulin M (IgM) monoclonal gammopathy is the most common paraproteinemic neuropathy, comprising a clinicopathologically and immunologically distinct entity. The clinical spectrum spans from distal paresthesias and mild gait imbalance to more severe sensory ataxia, with falls and a varying degree of distal sensorimotor deficits. In approximately 75% of patients, the monoclonal IgM immunoreacts with myelin-associated glycoprotein (MAG) and sulfoglucuronyl glycosphingolipid (SGPG), or other peripheral nerve glycolipids that serve as antigens. These antibodies are considered pathogenic because IgM and complement are deposited on the myelin sheath, splitting the myelin lamellae, while adoptive transfer of patients’ IgM into susceptible host animals causes sensory ataxia and reproduces the human pathology. In spite of the apparently convincing pathogenicity of these antibodies, the response to immunotherapies remains suboptimal. Clorambuscil, cladibrine, cyclophospamide and intravenous immunoglobulin may help some patients but the benefits are minimal and transient. Open-label studies in >200 patients indicate that rituximab is helpful in 30–50% of these patients, even with long-term benefits, probably by suppressing IgM anti-MAG antibodies or inducing immunoregulatory T cells. Two controlled studies with rituximab did not however meet the primary endpoint, mostly because of the poor sensitivity of the scales used; they did however show statistical improvement in secondary endpoints and improved clinical functions in several patients. This review provides an overview of the clinical phenotypes and immunoreactivity of IgM to glycolipids or glycoproteins of peripheral nerve myelin, summarizes the progress on treatment with rituximab as a promising therapy, discusses the pitfalls of scales used, identifies possible biomarkers of response to therapy and highlights the promising new anti-B cell or target-specific immunotherapies.

## Introduction

Benign monoclonal gammopathies, often called ‘monoclonal gammopathies of undetermined significance’ (MGUS), occur in up to 1% of normal people over the age of 50, with age-increasing incidence up to 1.7% above the age of 70 and 6% above 90.^1^ Monoclonal gammopathies are however 10 times more frequent in patients with polyneuropathy than age-matched controls, and 10% of adults with acquired polyneuropathy have a monoclonal gammopathy.^2,3^ If the gammopathies are categorized into immunoglobulin G (IgG), IgA and IgM subclasses, the incidence of polyneuropathy among patients with IgM monoclonal gammopathy can be as high as 50%, implying that 50% of patients with IgM MGUS may have or develop polyneuropathy.^3,4^ The association is not therefore fortuitous; it is also of importance to neurologists because paraproteinemic polyneuropathies comprise a potentially treatable group of autoimmune neuropathies, as proposed >35 years ago.^5^

Although paraproteinemic polyneuropathies can be seen in a setting of lymphoid malignancy such as myeloma, plasmacytoma, or Waldenström’s macroglobulinemia, most neuropathies recognized first by neurologists occur in connection with MGUS. The term MGUS was coined by hematologists to denote that the gammopathy is ‘of no significance’ when there is no hematological disease; in a patient with neuropathy however, MGUS is ‘of significance’ because, even if benign, the gammopathy may point to an immune association possibly amenable to immunotherapy.^5,6^

The patients who have a demyelinating polyneuropathy associated with IgG or IgA–MGUS behave like chronic inflammatory demyelinating polyneuropathy (CIDP) and the paraprotein is likely coincidental; in contrast, patients with polyneuropathy and IgM MGUS have distinct clinicopathologic and immunobiologic phenotypes. This review describes the clinical spectrum and highlights the progress in the immunopathogenesis and treatment of IgM–MGUS neuropathy, the most common paraproteinemic neuropathy.

## Clinical spectrum

**Figure 1.**
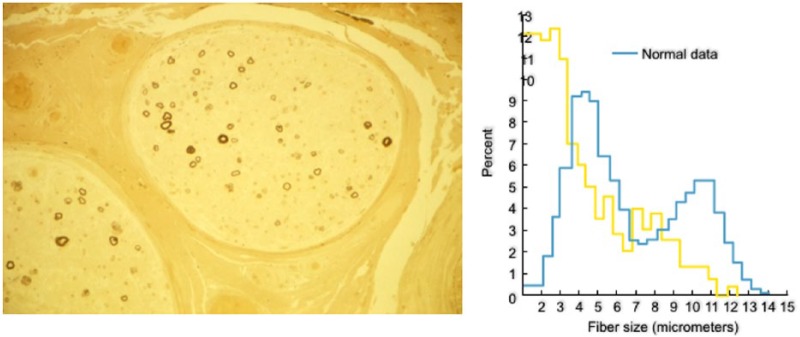
Cross-section of a sural nerve biopsy from a patient with immunoglobulin M antimyelin-associated glycoprotein antibody demyelinating polyneuropathy. The biopsy specimen is stained with toluidine blue and shows selective loss of large-size myelinated fibers (left); histogram (right) depicting the fiber-size distribution of the patient’s nerve (yellow), overlapped with the fiber-size distribution (blue) from normal data, clearly demonstrating the selective loss of large-size fibers.

Patients with IgM–MGUS typically manifest symptoms associated with dysfunction or loss of large myelinated fibers, most often presented with slowly progressive distal paresthesias, prominent in the feet, and impaired balance. The disease progresses very slowly, leading to impaired proprioception, sensory ataxia, unsteady gait, and a varying degree of distal muscle weakness, first seen in toe and foot extensors.^4–6^ A good number of patients have an action tremor prominent in the hands that is unrelated to impaired proprioception.^7,8^ The clinical spectrum varies substantially; some patients have only distal paresthesias and minimal gait imbalance that may not sufficiently affect daily activities or cause disability that could be captured with disability scales, as discussed;^9^ others manifest sensory ataxia and impaired gait; and still others have a combination of sensorimotor deficits with slowly progressive proximal and distal weakness that along with sensory ataxia, cause significant disability.^6,9,10^ Some patients may have subacute onset and faster disease course resembling CIDP with mixed features of demyelination and axonal loss.^11^ Immunofixation electrophoresis reveals an IgM monoclonal spike that remains present throughout the course, although at times becomes faint or temporarily undetectable; the total IgM may be normal, but is usually elevated three-to-five times above normal. The Cerebrospinal fluid (CSF) protein is often elevated. The IgM spike is also detected in the CSF and exhibits the same electrophoretic mobility as in the serum, in spite of the high-molecular weight of IgM, probably gaining access *via* the dorsal root ganglia that lack blood–CSF barrier or from a disrupted root–CSF barrier.^12^ Nerve-conduction studies demonstrate a uniformly slow conduction velocity with a rather characteristic prolonged distal motor and sensory latencies, indicative of distal demyelination.^13,14^ Sural nerve biopsy shows diminished numbers of myelinated axons with selective loss of large myelinating fibers, consistent with the clinical picture of impaired proprioception (Figure 1). Electron microscopy demonstrates a rather unique splitting of the outer myelin lamellae, probably linked to the presence of IgM deposits in the same area of the split myelin sheaths.^15^

### Clinical evaluation

The first goal is to ensure that the gammopathy is MGUS and not a sign of an underlying plasma cell dyscrasia, such as myeloma, plasmacytoma, or Waldenström’s macroglobulinemia. Monoclonal gammopathies are hematologically benign (MGUS) when the patients have: (a) <3 g/dl monoclonal protein in the serum; (b) no signs of renal insufficiency, osteolytic or osteosclerotic lesions in the bone survey, and signs of anemia or hypercalcemia; (c) no suppression of the uninvolved (IgG, IgA) polyclonal (background) Igs; and (d) stable amount of the monoclonal protein in follow-up examinations.^5,6,16^ When ominous signs of malignant plasma cell dyscrasia are detected, such as lytic bone lesions, Bence-Jones proteinuria, or progressively increased amount of monoclonal protein, a bone marrow examination is essential; the presence of >5% plasma cells and 12% lymphoid aggregates suggests malignancy. Because there is a 1% probability per year that MGUS may evolve into plasma cell dyscrasia,^1^ yearly follow-up examinations with immunofixation electrophoresis and Ig levels are necessary.^16^ Finding a free light chain should raise suspicion of amyloidosis derived from the variable region of the immunoglobulin light chain, mostly λ. Amyloid neuropathy is painful and often accompanied by autonomic symptoms, such as orthostatic hypotension, impotence, impaired gastric motility, or diarrhea. Diagnostically, deposits of amyloid may be found in the nerve and muscle biopsy, skin, abdominal fat or bone marrow.^16^

## Immunopathogenesis

### Antimyelin-associated glycoprotein antibodies

**Figure 2.**
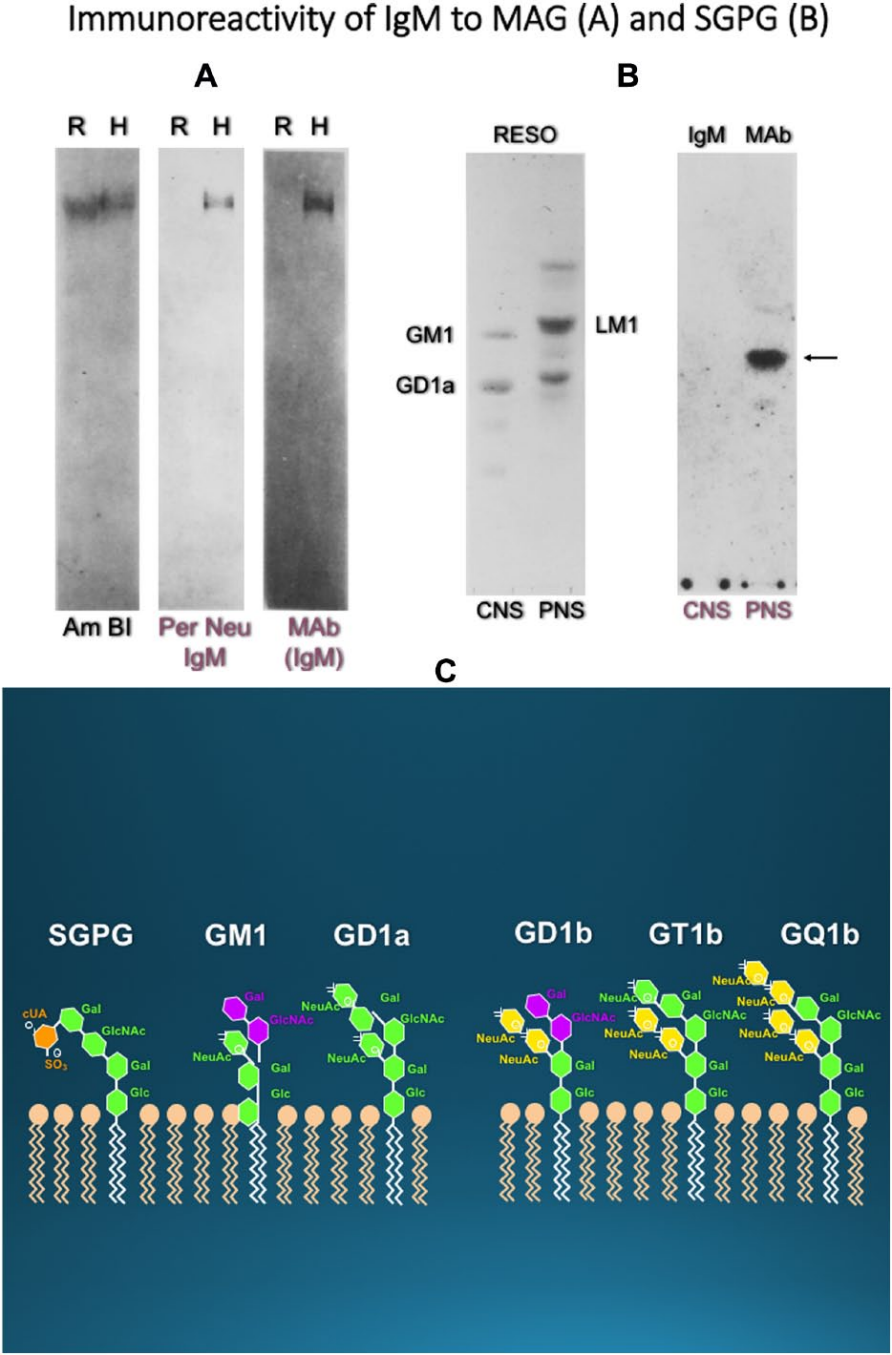
Immunoreactivity of patients’ immunoglobulin M to myelin-associated glycoprotein and glycolipids. (A) Immunoblots of sodium dodecyl sulfate gels with purified MAG extracted from rat (R) or human (H), are stained for protein with Am Bl (left) or immunostained with the serum of a patient with IgM MGUS neuropathy (middle lane). The patient’s IgM binds only to purified human MAG (middle lane; Per Neu); the reactivity is identical to the band obtained with a commercial MAb to human MAG (right) (from Dalakas^16^ and Ilyas et al^19^). (B) TLC overlay experiments show that the IgM from an anti-MAG-reacting patient recognizes glycolipid antigens of human peripheral nerve. The panel on the left shows resorcinol-stained ganglioside fractions isolated from human brain (CNS) or from human peripheral nerve (PNS). The prominent gangliosides are labelled for reference. The panel on the right shows an autoradiograph obtained from overlaying the same TLC plate with the patient’s serum followed by radiodinated antihuman IgM. The major glycolipid antigen recognized by the patients’ IgM (arrow) is SGPG found exclusively in the PNS. SGPG is between two minor glycolipid antigens (shown on the left gel) migrating above and below SGPG (from Dalakas^16^ and Ilyas et al.^19^). (C) Common glycolipids and their disialosyl moieties, consisting of NeuAca2-8NeuAc, that serve as antigens in autoimmune neuropathies The SGPG is most common in anti-MAG-neuropathy; the GM1 in MMN; the GD1a and GT1b with two sialic acid residues, and the GT1b immunoreact with 25% of MAG-reactive patients who have IgM-MGUS and ataxic neuropathy; the GQ1b ganglioside (with two disialosyl moieties) is the most specifc target antigen in Miller-Fisher syndrome (from Dalakas and Quarles^21^). MAG, myelin-associated glycoprotein; Am Bl, amido black; IgM, immunoglobulin M; MGUS, monoclonal gammopathies of undetermined significance; TLC, thin layer chromatography; MAb, monoclonal antibody; RESO, resorsinol; CNS, central nervous system; PNS, peripheral nervous system; SGPG, sulfoglucuronyl glycosphingolipid; GM1, bearing one sialic acid; MMN, Multifocal Motor Neuropathy; GD1a, bearing two sialic acids; GT1b, ganglioside with three sialic acids; GQ1b, ganglioside with four sialic acids.

The main breakthrough in this neuropathy was the discovery by Latov and colleagues of IgM immunoreactivity to myelin-associated glycoprotein (MAG), a 100 kDa glycoprotein of the central and peripheral nerve myelin.^17^ Sera from approximately 50% of these patients react with MAG or with acidic glycolipids that share antigenic determinants with MAG^10,16–19^ and reside in the carbohydrate component of the MAG molecule, as we have demonstrated by loss of reactivity after deglycosylation of purified MAG^18^ (Figure 2A). An important observation was the subsequent finding that the anti-MAG IgM coreacts with an acidic glycolipid in the ganglioside fraction of the human peripheral nerve, which by ion-exchange chromatography was seen between monosialotetrahexosylganglioside bearing one sialic acid (GM1) and bearing two sialic acids (GD1a) and identified as a SGPG^19^ (Figure 2B). In contrast to MAG, which is present in both, the peripheral nervous system (PNS) and the central nervous system (CNS), SGPG is found exclusively in the peripheral nerves. SGPG also recognizes the human natural killer-1 (HNK-1) trisaccharide epitope, present on both MAG and other peripheral nerve glycoconjugates.^20^

Human anti-MAG antibodies can be detected in the patients’ sera with enzyme-linked immunosorbent assay (ELISA) or Western blots by electrophoresing purified MAG, isolated myelin, or whole brain homogenates.^19,20^ Because sera positive for anti-MAG antibodies almost always recognize the SGPG glycolipid, the assay has been performed by certain laboratories using SGPG as antigen instead of human MAG. It is clearly preferable to use MAG instead of SGPG because the IgM antibodies bind to MAG 10–100 times more strongly than to SGPG; consequently, low-affinity anti-MAG antibodies may be missed if SGPG is used as antigen.^16,19,20^ In a large series of patients’ samples, the presence of MAG antibodies detected by ELISA was the most preferable assay because it was sensitive and easier than Western blotting.^22^

### Other antiganglioside antibodies

In some patients with neuropathy clinically identical to IgM-anti-MAG phenotype, the serum may not react with MAG or SGPG, but only with various gangliosides most commonly those containing either a disialosyl moiety (e.g. GD1b, GQ1b, GT1b, the GalNac-GM1b, or Gal-NAc-GD1a) or two gangliosides that share epitopes with GM2, a combination of GM2 and GM1, or GM1 and GD1b.^21,23–25^ Overall, more than half of the IgM paraproteins recognize MAG and SGPG, and 75% of the rest recognize ganglioside antigens, indicating that acidic glycolipids are the most common antigenic epitopes in immune-mediated neuropathies, as depicted in Figure 2C.^21,23–26^

### Causal relationship between antimyelin-associated glycoprotein/sulfoglucuronyl glycosphingolipid antibodies and the neuropathy

Because MAG is present only in small amounts in the peripheral nerves, compared with its abundance in the CNS (which is spared in these patients), the immunoreactivity of IgM to MAG was considered by some as a nonpathogenic, binding to antigens with carbohydrate epitopes. The discovery of PNS-specific SGPG glycolipid however, that all anti-MAG-positive patients immunoreact with, has strengthened the view that glycolipids, along with glycoproteins, may be the primary antigenic targets.^9,10,16,26,27^ The following factors provide convincing evidence that these antibodies are causally related to the neuropathy:

(1) Sural nerve biopsies demonstrate deposition of IgM and complement on the myelinated nerve fibers,^28^ suggesting that activated complement is needed to induce demyelination.

(2) The patients’ IgM recognizes neural cell adhesion molecules and co-localizes with MAG on the split myelin lamellae,^15,16,26,27,29^ implying involvement of IgM in myelin disadhesion. This view is strengthened by observations on the patients’ skin biopsies which showed deposits of IgM, complement C3d, and MAG on the dermal myelinated fibers associated with reduction of intraneural nerve fiber density.^29^

(3) The patients’ whole serum or purified IgM, when injected with fresh complement into feline peripheral nerves, causes complement-dependent demyelination and conduction block within 2–9 days.^30^

(4) Systemic administration of anti-MAG IgM paraprotein in chickens, causes segmental peripheral nerve demyelination with deposition of IgM on the outer myelin lamellae and splitting of the myelin sheath in a pattern similar to the one observed in the human neuropathy.^31^

(5) Immunization of cats with purified SGPG causes ataxic neuropathy, clinically resembling the anti-MAG/SGPG sensory ataxic phenotype, with inflammation within the dorsal root ganglionic neurons.^32^

## Treatment

In patients with IgG or IgA MGUS who have an axonal neuropathy, the paraprotein is probably of unknown significance; if their neuropathy however is demyelinating, it should be treated as CIDP. In these patients, a causal relationship between the IgG or IgA MGUS and the neuropathy has not been established because in contrast to IgM-MGUS, the A and G immunoglobulins do not recognize MAG or other antigens, except rarely when they recognize complex glycolipids, as shown in one of our patients with IgA gammopathy.^33^

Since the IgM-anti-MAG antibodies appear to exert a pathogenic effect on the myelin structure and function,^34,35^ it was expected that suppressing their production, removal of the antibodies or modification of the immune network should have a therapeutic effect. Unfortunately, this is not the case in clinical practice because conventional immunotherapies and chemotherapies offer marginal or transient benefit,^26,27,35–42^ necessitating the need for more specific therapies.

### When to initiate therapy

The aforementioned clinical spectrum and disease severity should be taken into account in therapeutic decisions. Patients who only have distal paresthesias without gait difficulties that affect daily activities, should be managed with supportive therapy consisting of exercise and balance training. If, however, manifest gait ataxia, falls and muscle weakness, or present with a CIDP-like picture from the outset, they should be treated early before permanent deficits due to axonal degeneration take place.^36–42^

### Chemotherapeutic agents

Chlorambucil and prednisone, a combination used to treat Waldenström’s macroglobulinemia, can improve or stabilize a small number of patients, as we have witnessed in one,^43^ but the benefit is minimal and does not often outweigh the risks.^37–42^

Oral cyclophosphamide, in a randomized controlled trial of 35 patients (500 mg for 4 days) in combination with 60 mg prednisolone, every 4 weeks for 6 months, showed some improvements in the Medical Research Council (MRC) sumscores,^44^ but methodological problems with the study design and malignant transformation observed in 10% diminished the impact of the outcome.^44^

Fludarabine, applied to 16 patients in an open-label study, showed transient benefits in some patients^45,46^ and significant adverse effects in 4.

### Intravenous immunoglobulin

In three anti-MAG-positive patients we treated with intravenous immunoglobulin (IVIG),^47^ an improvement in motor and sensory functions was observed, prompting us to conduct the first controlled study. Among 11 randomized patients, the neuropathy scores improved in three patients; the overall differences between IVIG and placebo were not however significant.^48^ A small multicenter study conducted in Europe, in spite of some methodological issues, showed modest benefits that were statistically significant only for the secondary outcome measures.^49,50^ In an uncontrolled study in patients with sensory ataxic neuropathy associated with anti-GD1b, but not anti-MAG, antibodies, IVIG showed short-term benefits.^51^ Overall, the benefits from IVIG have been minimal.^50^

### Rituximab: a promising anti-B-cell therapy for antimyelin-associated glycoprotein neuropathy

Rituximab is a monoclonal antibody against CD20, a B-cell surface antigen present on pre-B cells and throughout the B-cell life cycle, but not on plasma cells.^52–54^ Rituximab causes depletion of circulating B cells, prompting several trials.

Uncontrolled studies. First tried in a rather heterogeneous group of 21 patients, rituximab improved most patients 1 year after treatment and reduced IgM autoantibodies.^55,56^ In another, prospective, open-label study, six of nine patients improved after 12 months with improved electrophysiology and reduced anti-MAG antibodies.^57^ Using double the standard dose (750 mg/kg every week for 1 month, instead of 375 mg/kg), the same authors noted improvement in four of eight additional patients.^58^ Cumulative data from approximately 200 patients treated in the last 15 years show that rituximab helped 30–50% of the patients.^36,59–63^ In most, IgM levels decreased by 39% and anti-MAG by 68%.^60^

**Figure 3.**
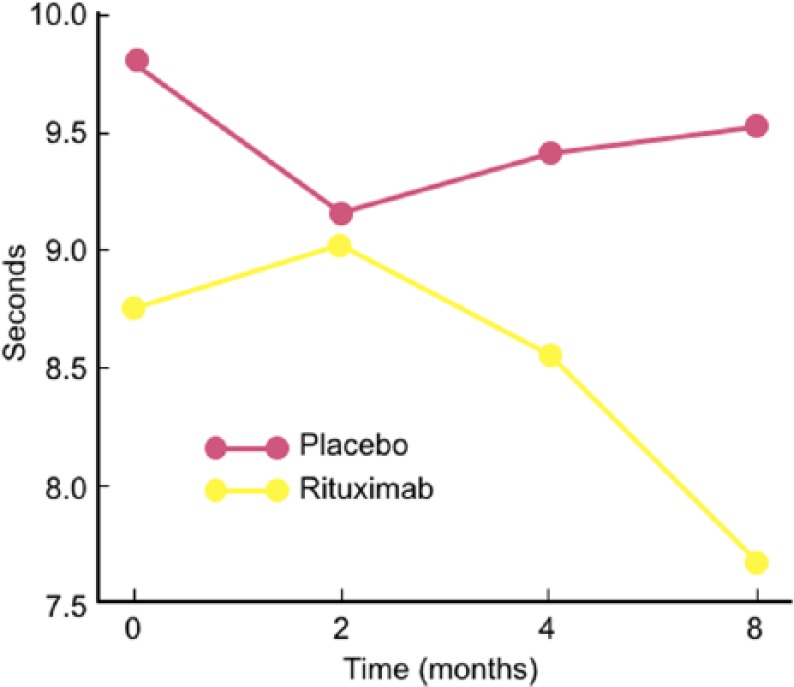
Effect of rituximab in the time to 10 m walk in a double-blind study.^64^ Difference in the 10 m walk time by repeated-measures analysis of variance between the rituximab and placebo groups. A significantly improved gait was seen in the rituximab group (yellow) compared with placebo (*p* = 0.042) (red) in the intention-to-treat analysis.

**Figure 4.**
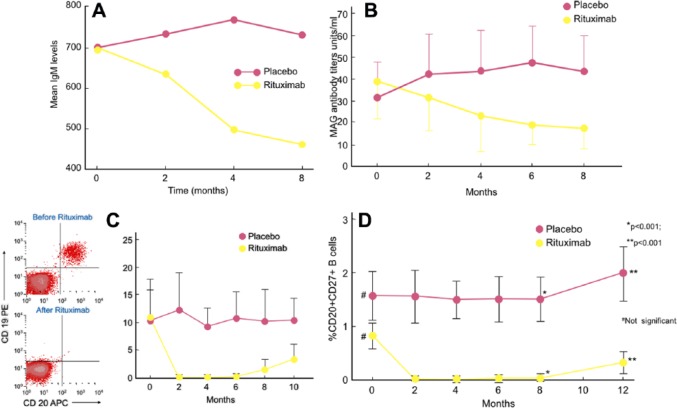
Reduction of B cells, immunoglobulin M, and antimyelin-associated glycoprotein after rituximab in a controlled study. (A, B) The IgM levels are decreased by 34% (A) and the anti-MAG antibody titers by >50% (B), most prominently at month 8, in rituximab-treated patients compared with placebo; (C, D) Kinetics of depletion and reappearance of CD20+ B cells (C) and the CD20+ CD27+ memory B cells (D) in rituximab-treated patients compared with placebo. B cells are depleted in the periphery by month 2 and start rebounding by month 6; the CD20+ CD27+ memory B cells start reappearing a little later by month 8 (D), often consistent with the need for reinfusion if there are early signs of clinical relapse.^64^ IgM, immunoglobulin M; MAG, myelin-associated glycoprotein; APC, antigen-presenting cell, PE, PRE (pre-B cells).

Controlled studies. In spite of the above promising observations, two controlled studies, using different scales and endpoints, have not convincingly captured this benefit. In the first double-blind placebo-controlled study of 26 patients, 13 were randomized to rituximab and 13 to placebo.^64^ The primary endpoint was a change of >1 in Inflammatory Neuropathy Cause and Treatment (INCAT) leg disability score at month 8. By intention-to-treat (ITT), 4/13 (30%) of rituximab-randomized patients improved by >1 INCAT score, compared with 0/13 placebo-receiving patients (*p* = 0.096). One of the patients randomized to rituximab however, was found to have a normal INCAT score at entry and could not improve; if this patient is excluded, the results become significant (*p* = 0.036). The time to 10 m walk, a secondary endpoint, was significantly reduced in the rituximab-randomized group by ITT (Figure 3). On clinical grounds, walking and daily activities improved in 7/13 (53.8%) rituximab-treated patients, compared with 0/13 placebo-treated ones, reaching a percentage similar to the one observed in cumulative uncontrolled series, as mentioned earlier. The serum IgM was reduced by 34% and the anti-MAG titers by 50%^64^ (Figure 4A–B). In this series, the most improved patients were those who had high anti-MAG titers and most severe sensory deficits. The main drawback of the study was the scales used; INCAT is a disability motor scale and could not effectively capture small functional changes in sensory function, especially mild gait impairment and sensory ataxia.

Important useful data regarding the immunobiology of B cells and antibody production were observed in clinically-improved patients. Because the signs of clinical response coincided with depletion of B cells and reduction of anti-MAG antibodies (Figure 4A–C), rituximab (that does not affect plasma cells), has likely depleted the memory B cells, which are the precursors of short-lived plasma cells. Indeed, the memory CD20+ CD27+ B cells were depleted 1 month after rituximab infusion and remained undetectable for 6 months; they started reappearing by month 8 and coincided with the slow rise of IgM serum level (Figure 4A–D). Study of B cell Ig gene usage between the four responders compared with nonresponders, revealed clonal expansions of circulating IgM memory B cells that recognized MAG antigen.^65^ Most importantly, in the nonrituximab responders, the load of IgM memory B cells and the CDR3 clonally expanded sequences were higher and persisted before and after therapy, a finding we interpreted to suggest that low efficiency to reduce B-cell expansions may be associated with poor clinical response.^65^ Accordingly, higher doses of rituximab (as shown in patients who improved with double dosage),^58^ or more potent B-cell-depleting agents that reduce further the clonally related autoreactive B cells may be more effective.^36^ A significant increase of Foxp3-positive immunoregulatory T cells was also observed at 8 months, suggesting that rituximab affected the immunoregulatory T-cell balance.^64^

A second, much larger controlled study, was conducted in 54 patients, 26 randomized to rituximab and 28 to placebo.^66^ Using sensory scales as primary endpoint at month 12, the authors did not capture any benefit; they did, however, observe a significant response in secondary endpoints based on disability and time-to-walk scales.^67^ The discrepancy between the two well-conducted clinical trials is obviously related to patient selection and scales used, highlighting the inadequacy of sensory INCAT to capture and quantify small, but clinically meaningful, changes related to gait ataxia.

### Possible biomarkers of patient subsets responding to rituximab

Obviously, the question is not anymore whether rituximab is helpful or not, but why it benefits <50% of the patients and whether there are biomarkers predicting response.^36^ Demyelinating pattern, absence of treatment and older age were significant risk factors for disability worsening,^62^ while predominantly motor deficits and subacute progression were associated with positive response.^11^ Gender, ataxia, tremor and IgM anti-MAG antibody titers were observed to be irrelevant in predicting response;^11,60,62^ in one study, recurrence of disease correlated best with high baseline anti-MAG titers and increasing anti-MAG antibodies at follow up.^63^

Avidity, and variability in binding of anti-MAG/SGPG antibodies to target antigens, is a factor possibly explaining different therapeutic responses.^68^ Serum levels of BAFF (B-cell activating factor), a key costimulatory molecule for B-cell survival, proliferation and immunoglobulin production,^52,54^ were higher in anti-MAG patients not responding to rituximab;^61^ BAFF increased post rituximab in those who responded but, in those who relapsed, BAFF returned to baseline, suggesting that BAFF may be useful in predicting responses and relapses.^61^ The finding that therapeutic efficacy depends on reconfiguration of memory B cells through sustained reduction of autoreactive clonal expansions,^65^ could also explain why rituximab is effective in only a patient subset. The need for retreatment to maintain stability in patients with prior response to rituximab may be monitored by the B27-memory B cells that start re-emerging after 8 months^64^ (Figure 4D).

### Safety profile

Notwithstanding the benefit of rituximab for certain patient subsets, potential side effects should be considered.^36,52,54^ Rituximab may at times worsen anti-MAG neuropathy,^69,70^ a phenomenon we have also witnessed in three patients; this is probably related to immunomodulatory imbalance and release of proinflammatory cytokines that could possibly change the permeability of the blood–nerve barrier allowing the influx of IgM antibodies within the nerve.^36,70^ This worsening is rare or self-limited and is almost always reversed by IVIG. Rituximab has an excellent safety profile, but infections from common bacteria or viral agents can occur.^36,52–54^ Progressive multifocal leukoencephalopathy has been reported in some patients with B-cell malignancies receiving rituximab plus chemotherapy (R-CHOP, fludarabine).^36,52,54,71^ Vigilance is recommended, especially in patients receiving concomitant immunosuppressants.

## The future: new drugs, better scales, and antigen-specific therapies

### Anti-B-cell agents

If rituximab helps 30–50% of anti-MAG neuropathy patients, the newer anti-B-cell agents that cause more profound or sustained B-cell depletion, could theoretically be more encouraging.^36,52,54^ B cells not only affect complement activation and antibody production but are strong antigen-presenting cells affecting autoregulation and T-cell activation,^52,54^ which explains the benefit of rituximab to diseases with dominant T-cell receptor gene usage.^54,72^ Occrelizumab, the humanized version of rituximab that induces more complement activation, is approved for multiple sclerosis and should be considered. Ofatumumab that targets two different epitopes on CD20 causes more significant B-cell depletion; it appears promising in multiple sclerosis (MS) and could be another candidate drug. Obinituzumab, a humanized monoclonal against CD20 approved for chronic lymphocytic leukemia, causes profound B-cell depletion; the drug, recently tried in two anti-MAG neuropathy patients resistant or partially responding to rituximab did not show efficacy, in spite of reducing the IgM levels and anti-MAG titers.^73^ Belimumab against the B-lymphocyte stimulator BLyS, approved for lupus, may be another alternative, although its failure in MS and myasthenia gravis, lowers its priority status.^36,54^

### Scales

Because the disease exhibits a wide clinical spectrum, future studies should include patients with homogeneous clinical phenotypes and disease duration. Patients with very slowly progressing disease develop secondary axonal changes over time and worsening ataxia, with loss of ganglionic neurons; as a result, these patients are less likely to respond compared with patients having more recent disease and subacute progression.^11^ Applying functional scales to capture even minor impairments in life activities, gait imbalance and hand tremors is fundamental. For example, in our controlled study,^64^ more patients were able to perform additional daily activities after rituximab compared with placebo, experienced steadier and faster walking, better balance, easier use of utensils, and fewer falls, pain or paresthesias; none of these changes could be however numerically captured with the INCAT disability scales.^64^

### Hand tremor

The tremor seen in many anti-MAG patients can be incapacitating. It is a neuropathic tremor, unrelated to impaired proprioception or a coexisting cerebellar disease; it is unresponsive to conventional antiessential tremor drugs but can improve in some patients responding to immunotherapy. Unilateral thalamic deep brain stimulation (DBS) applied in one patient unresponsive to plasma exchange, cyclophosphamide, steroids, primidone, and gabapentin, induced sustained improvement.^73^ DBS interferes with cerebellar circuits implicated in tremor generation; because in anti-MAG patients the cerebellum may receive disordered sensory input,^75^ DBS needs further study.

### Antigen-specific therapy

Anti-MAG antibodies are thought to interfere directly with MAG’s involvement in adhesion and signaling processes at the axon–glia interface^76^ resulting in cellular disadhesion. Because MAG has a higher HNK-1 epitope density than other myelin glycoconjugates leading to strong IgM antibody binding, removal of HNK with synthetized glycopolymers may prevent binding of anti-MAG IgM to human MAG.^76^ On this basis, in a mouse model for anti-MAG neuropathy, the glycopolymer PL84(mimHNK-1), designed as an autoantibody scavenger by mimicking the natural HNK-1 glycoepitope, effectively removed the pathogenic anti-MAG antibodies, offering promising potential for antigen-specific immunotherapy.^77^
